# Mediterranean Diet Adherence and Cognitive Function in Older Adults: Heterogeneity in the Association Across BMI Categories

**DOI:** 10.1155/ije/7060250

**Published:** 2026-10-28

**Authors:** Zhaoxing Liu, Mengchen Qin, Chongye Zhao, Ji Ding, Yongkang Li, Weidong Cheng

**Affiliations:** ^1^ School of Traditional Chinese Medicine Southern Medical University Guangzhou Guangdong China fimmu.com; ^2^ Department of Biostatistics School of Public Health Southern Medical University Guangzhou Guangdong China fimmu.com

**Keywords:** body mass index, cognitive function, Mediterranean diet, older adults

## Abstract

Cognitive decline is a major public health concern among older adults, and both dietary quality and adiposity have been associated with cognitive health. Using data from the 2011–2014 National Health and Nutrition Examination Survey, we conducted a cross‐sectional analysis of 2492 U.S. adults aged ≥ 60 years. Adherence to the Mediterranean diet was quantified using the Mediterranean diet index (MEDI) derived from two 24‐h dietary recalls, and body mass index (BMI) was classified into four categories: underweight, normal weight, overweight, and obesity. Global cognitive function was computed by standardizing and summing scores from the Consortium to Establish a Registry for Alzheimer’s disease immediate and delayed recall tests, the animal fluency test, and the Digit Symbol Substitution test. After multivariable adjustment, higher MEDI scores were associated with better global cognitive function, with positive associations also observed for verbal fluency and processing speed. Restricted cubic spline analysis supported a positive, approximately linear dose–response association between MEDI score and global cognitive function. However, in the linear interaction analysis, this association differed across BMI categories. Among normal‐weight participants, higher MEDI scores were associated with better global cognitive function. Underweight status was associated with poorer immediate and delayed recall, whereas overweight status was associated with lower verbal fluency. Overall, greater adherence to a Mediterranean dietary pattern was associated with better cognitive performance, particularly verbal fluency and processing speed, among older U.S. adults. BMI‐related heterogeneity in this association supports prospective longitudinal studies and intervention trials incorporating repeated assessments of diet, adiposity, metabolic health, and cognition.

## 1. Introduction

Cognitive decline in later life is an increasingly important public health concern because it can undermine functional independence. An estimated 57.4 million people were living with dementia worldwide in 2019, and this number is projected to reach 152.8 million by 2050 [1]. Among individuals who develop dementia, declines in memory, attention, and executive function often emerge years before diagnosis, providing a potential window for risk‐reduction interventions [2]. The 2024 Lancet Commission identified 14 potentially modifiable risk factors across the life course, suggesting that addressing these factors could prevent or delay nearly half of dementia cases [3]. More recently, the U.S. Study to Protect Brain Health Through Lifestyle Intervention to Reduce Risk (U.S. POINTER) randomized clinical trial found that, among older adults at increased risk of cognitive decline and dementia, a structured multidomain lifestyle intervention led to a modestly greater improvement in global cognition than a self‐guided intervention [4]. However, the relationships between modifiable lifestyle factors and cognitive function or dementia risk, and whether these associations vary across subgroups of older adults, remain incompletely understood.

Diet is a modifiable lifestyle factor with implications for both metabolic health and cognitive aging [5]. The Mediterranean dietary pattern, characterized by a high intake of plant foods, fish, and unsaturated fats and a low intake of red and processed meat, is among the most extensively studied healthy dietary patterns [5, 6]. Prospective studies, including analyses from the U.K. Biobank and other population‐based cohorts, have associated greater adherence to the Mediterranean diet with a lower risk of dementia and more favorable cognitive trajectories [7–9]. However, findings from randomized trials of cognitive outcomes have been inconsistent. A cognitive substudy of the Prevención con Dieta Mediterránea (PREDIMED; Prevention with the Mediterranean Diet) trial reported beneficial effects on global cognition and selected cognitive domains [10]. By contrast, a 3‐year randomized trial of the related but distinct Mediterranean–Dietary Approaches to Stop Hypertension Intervention for Neurodegenerative Delay (MIND) diet found cognitive improvement in both groups but no significant between‐group difference [11]. Differences in dietary assessment, baseline nutritional status, comparator diets, cognitive measures, and follow‐up duration may partly account for these inconsistent findings [5].

Adiposity and related metabolic disturbances have also been linked to cognitive health, although these associations vary across the life course [12–14]. Higher body mass index (BMI) and central adiposity in midlife have been associated with an increased risk of cognitive decline and dementia later in life [12, 13]. Metabolic syndrome has also been associated with smaller brain volumes, a greater burden of white matter hyperintensities, and poorer performance across multiple cognitive domains in adults without dementia [14]. In older adults, however, these associations are more difficult to interpret. Pooled longitudinal data indicate that BMI may decline during the preclinical phase of dementia [12], and large population studies have reported higher dementia rates among individuals with low BMI [15]. These observations suggest that the effects of obesity on cognition in older adults should be interpreted in the context of metabolic health and age‐related changes in nutritional status.

Several studies have examined the association between Mediterranean diet adherence and cognitive outcomes in older adults with overweight or obesity. In a prospective analysis of 6647 participants in the PREDIMED‐Plus cohort, greater baseline adherence to the Mediterranean diet was associated with more favorable 2‐year changes in a general cognitive screening measure and in measures of attention, processing speed, and cognitive flexibility [16]. In a smaller 3‐year cognition substudy from the same trial, greater adherence to an energy‐restricted Mediterranean diet was associated with greater improvements in memory, and memory gains were also correlated with reductions in BMI [17]. A subsequent 6‐year analysis of 746 PREDIMED‐Plus participants linked greater adherence to slower global cognitive decline; a Mediterranean diet‐related gut microbial signature was also associated with more favorable cognitive trajectories [18]. Findings from other populations, however, have not been uniformly positive. In the randomized Building Research in Diet and Cognition (BRIDGE) trial of older adults with obesity, Mediterranean diet interventions with and without a weight‐loss component improved dietary adherence, and the weight‐loss intervention reduced body weight, but neither intervention improved cognition relative to the usual‐diet control over 14 months [19]. Because these studies were restricted to participants with overweight or obesity, they could not determine whether the association between Mediterranean diet adherence and cognition differs across the broader range of BMI categories. Evidence directly comparing underweight, normal‐weight, overweight, and obesity groups in a general older population therefore remains limited.

The present cross‐sectional study used nationally representative data from the 2011–2014 National Health and Nutrition Examination Survey (NHANES) to evaluate the association of a Mediterranean diet index (MEDI) score derived from two 24‐h dietary recalls with global cognitive function and four domain‐specific measures: immediate recall, delayed recall, verbal fluency, and processing speed, among U.S. adults aged 60 years or older. We further evaluated the associations of BMI category with cognitive outcomes after multivariable adjustment, characterized the functional form of the association between MEDI score and global cognitive function using restricted cubic splines (RCS) and exploratory piecewise linear regression, and formally tested the interaction between MEDI score and BMI category. Additional subgroup analyses evaluated potential heterogeneity across demographic and clinical characteristics, and a sensitivity analysis examined central obesity defined by waist circumference as an alternative measure of adiposity. The primary objective was to evaluate the association between MEDI score and cognitive function and to determine whether this association differed across BMI categories.

## 2. Methods

### 2.1. Study Population

NHANES is an ongoing, cross‐sectional program designed to evaluate the health and nutritional status of the noninstitutionalized civilian population in the United States [20]. Administered by the National Center for Health Statistics and supported by the Centers for Disease Control and Prevention, NHANES employs a complex, multistage probability sampling design and collects data in 2‐year cycles through structured household interviews and standardized physical examinations conducted at mobile examination centers (MEC), where biological specimens, including blood and urine, are obtained. All survey protocols are reviewed and approved by the National Center for Health Statistics Research Ethics Review Board, and written informed consent is obtained from all participants. The data are publicly available, and detailed methodological information is provided in the official survey documentation.

For the present analysis, data from the 2011–2012 and 2013–2014 NHANES cycles were combined. Among 19,931 participants initially identified, individuals younger than 60 years and those with missing cognitive assessment data were excluded, resulting in a final analytic sample of 2492 participants (Figure 1). Eligibility required complete cognitive data from the Consortium to Establish a Registry for the Alzheimer’s Disease Word Learning (CERAD‐WL) test, the animal fluency test (AFT), and the Digit Symbol Substitution test (DSST). Participants were also required to have complete measured BMI data for BMI categorization, as well as complete dietary data necessary for calculating Mediterranean diet adherence scores.

**FIGURE 1:**
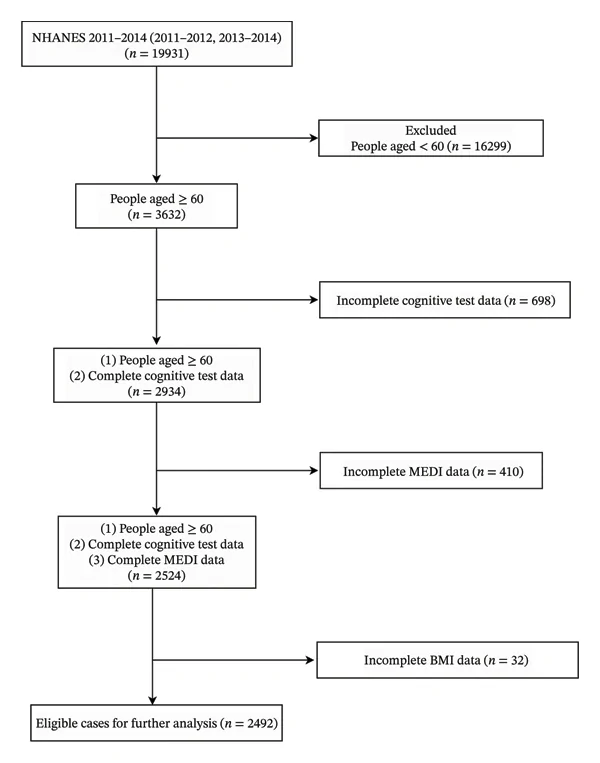
Flowchart of the study population. MEDI, Mediterranean diet index; BMI, body mass index.

### 2.2. Variable Definitions

#### 2.2.1. Cognitive Function

Cognitive function was assessed among participants aged 60 years and older during the 2011–2014 survey cycles. All cognitive assessments were administered by trained interviewers through face‐to‐face interviews conducted at the MEC. The assessment battery included the CERAD‐WL test, the AFT, and the DSST. The CERAD‐WL assesses immediate and delayed verbal memory and consists of three consecutive learning trials that together constitute the immediate recall test (IRT; score range, 0–30), followed by a delayed recall trial administered approximately 8–10 min later as the delayed recall test (DRT; score range, 0–10). The AFT assesses categorical verbal fluency and semantic memory by asking participants to name as many animals as possible within 1 minute, with performance quantified as the number of correct responses, reflecting aspects of executive function and semantic retrieval. The DSST, a subtest of the Wechsler Adult Intelligence Scale–Third Edition, measures processing speed, sustained attention, and working memory by requiring participants to match symbols to numbers according to a reference key within 2 min, producing scores from 0 to 105. To derive a measure of global cognitive function, raw scores from CERAD IRT, CERAD DRT, AFT, and DSST were standardized to Z‐scores (mean, 0; standard deviation, 1) and summed to create a composite score, with higher values indicating better overall cognitive performance [21].

#### 2.2.2. BMI Category

BMI values were obtained from the Body Measures data files of the NHANES examination component. Participants were classified into four mutually exclusive categories: underweight (< 18.5 kg/m^2^), normal weight (18.5 to < 25.0 kg/m^2^), overweight (25.0 to < 30.0 kg/m^2^), and obesity (≥ 30.0 kg/m^2^) [22].

### 2.3. Dietary Intake Assessment

#### 2.3.1. Dietary Data Collection

Dietary intake data were obtained from two 24‐h dietary recall interviews administered in NHANES using the United States Department of Agriculture (USDA) Automated Multiple‐Pass Method [23]. The first recall was conducted in person at the MEC, and the second recall was completed by telephone 3–10 days later. For each recall day, all foods and beverages consumed during the preceding 24‐h period from midnight to midnight, including the type and amount of each reported item, were recorded. To approximate usual dietary intake, data from the two recalls were averaged when both recall days were available. For dietary pattern analysis, reported foods and beverages were linked to USDA Food Patterns Equivalents Database (FPED)–derived food pattern components [24, 25].

#### 2.3.2. Construction of the MEDI

Adherence to the Mediterranean dietary pattern was assessed using the MEDI, operationalized in the present study using the dietary index framework based on the PREDIMED dietary pattern approach [6, 25]. For each of the two NHANES 24‐h dietary recalls, reported foods and beverages were linked to FPED‐derived food pattern components and converted into standardized serving‐size equivalents for MEDI scoring. The MEDI comprised 11 dietary components, including seven positively scored components (olive oil, vegetables, fruits, legumes, nuts, fish or seafood, and moderate alcohol consumption) and four reverse‐scored components (discretionary fats, sweets, sugar‐sweetened beverages, and red and processed meats), with component scores assigned according to predefined intake criteria for each recall day. Component scores were first assigned for each recall day according to the predefined MEDI criteria, and the two recall‐specific component scores were then averaged when both recall days were available; therefore, intermediate values (e.g., 0.5) could occur in the final component score after averaging across the two recall days [25]. Because the present study used two nonconsecutive 24‐h dietary recalls rather than the Mediterranean Diet Adherence Screener (MEDAS), intake amount was captured directly from the reported quantity of each recalled food or beverage, whereas frequency was not measured as habitual long‐term intake frequency in the same way as MEDAS. Instead, it was approximated by repeated short‐term dietary assessment across two recall days and by averaging day‐specific component scores. Thus, the derived MEDI should be interpreted as a recall‐based estimate of Mediterranean diet adherence rather than a direct MEDAS measure [26]. Although total energy intake was available in the NHANES dietary data, it was not treated as an independent MEDI scoring component in the present analysis. The total MEDI score ranged from 0 to 11, with higher scores indicating greater adherence to the Mediterranean dietary pattern.

### 2.4. Covariates

Relevant covariates were included in the analyses based on prior evidence linking cognitive function with dietary quality. Demographic characteristics encompassed age, sex, race, education, and the poverty–income ratio (PIR). Lifestyle and health‐related factors included smoking status, categorized as never, former, or current, and alcohol consumption frequency, classified as nondrinker, 1 to 5 times per month, 5 to 10 times per month, or more than 10 times per month. In addition, diabetes, depression, systolic blood pressure (SBP), diastolic blood pressure (DBP), stroke, and cardiovascular disease (CVD) were considered potential confounders. Stroke and CVD were ascertained from self‐reported physician diagnoses, with CVD defined as congestive heart failure, coronary heart disease, angina pectoris, or myocardial infarction [27].

### 2.5. Statistical Analysis

All analyses were conducted in accordance with NHANES analytical guidelines and incorporated the appropriate dietary sample weights (WTDR2D) to account for the complex, multistage probability sampling design [28]. Continuous variables were presented as medians with interquartile ranges (IQRs), and categorical variables were expressed as frequencies and percentages [n (%)]. Group differences were assessed using the Mann–Whitney U test for comparisons between two groups and the Kruskal–Wallis H test for comparisons involving three or more groups.

To examine the associations of MEDI scores and BMI categories with global cognitive function, weighted multivariable linear regression models were constructed, with normal weight serving as the reference category for BMI. The unadjusted model included no covariate adjustment. Model 1 was adjusted for age, sex, and race. Model 2 was further adjusted for education, PIR, smoking, SBP, DBP, CVD, stroke, depression, diabetes, and BMI/MEDI (with additional mutual adjustment for BMI category or MEDI score, as appropriate). Multicollinearity in the fully adjusted model was evaluated using variance inflation factors (VIF), leading to the exclusion of alcohol and waist circumference to maintain model stability. Parallel analyses were conducted for domain‐specific cognitive outcomes, including the IRT, DRT, AFT, and DSST.

To further examine potential nonlinear dose–response relationships, RCS terms with three knots were incorporated into multivariable ordinary least squares (OLS) regression models. Models were adjusted for the covariates included in Model 2, with the stratification variable omitted from the corresponding stratum‐specific model, as appropriate. Potential effect modification by sex and BMI category was assessed separately by including interaction terms between the full RCS basis for MEDI score and the corresponding stratification variable. Overall spline interactions and nonlinear interaction components were evaluated separately, and the overall and nonlinear associations were also examined within each stratum. Stratum‐specific RCS models were subsequently fitted to characterize dose–response patterns and evaluate the overall and nonlinear associations within each stratum. Piecewise linear regression was used to explore potential threshold effects and estimate inflection points, and model fit was compared with that of the conventional linear model using likelihood ratio tests.

Potential effect modification by BMI category was examined using multivariable linear regression models. In the overall sample, interaction terms between continuous MEDI score and BMI category were included in the model. The overall *p* value for interaction was obtained using a partial F test comparing nested models with and without these interaction terms. Separate multivariable linear regression models were subsequently fitted within each BMI category to estimate adjusted β coefficients and 95% confidence intervals (CIs) for the association between MEDI score and global cognitive function. Models were adjusted for the same covariates as Model 2, with BMI category omitted from the BMI‐stratified models. In addition, subgroup analyses were conducted to evaluate the consistency of the association across demographic and clinical strata. Within each stratum, survey‐weighted multivariable linear regression models were used to estimate adjusted β coefficients and 95% CIs, with adjustment for the covariates included in Model 2 and exclusion of the corresponding stratification variable, as appropriate. Effect heterogeneity was assessed by including interaction terms between MEDI score and each subgroup variable in separate overall models.

All statistical analyses were performed using R software (Version 4.4.2). Statistical significance was defined as a 2‐tailed *p* < 0.05.

## 3. Results

### 3.1. Basic Characteristics of the Study Population

A total of 2492 participants were enrolled in the final analysis (Figure 1). The study population had a median age of 68 years, with median values of 28.1 kg/m^2^ for BMI, 101.2 cm for waist circumference, and 3.5 for the MEDI. Median scores were 19 (QR, 16–22) for the IRT, 6 (IQR, 5–8) for the DRT, 16 (IQR, 13–20) for the AFT, and 47 (IQR, 35–59) for the DSST. Scores on all four cognitive measures differed significantly across age groups and education levels and by stroke, diabetes, and CVD (all *p* < 0.05). IRT, DRT, and DSST scores differed by sex, whereas AFT scores did not. AFT and DSST scores differed by alcohol consumption; IRT and DSST scores, by smoking status; and IRT, AFT, and DSST scores, by depression status. IRT and DRT scores also differed across BMI categories (Table 1).

**TABLE 1:** General characteristics of participants and cognitive function tests.

Variables	Overall	IRT	DRT	AFT	DSST	
	*N* = 2492	19 (16–22)	6 (5–8)	16 (13–20)	47 (35–59)	
Sex						
Male	1199 (48%)	18 (15–21)	6 (4–7)	16 (13–20)	44 (33–55)	
Female	1293 (52%)	20 (17–23)	7 (5–8)	16 (13–20)	50 (37–62)	
		**< 0.001**	**< 0.001**	0.333	**< 0.001**	*p*‐value
Age (years)	68.0 (63.0, 75.0)					
Age group						
60–69	1358 (54%)	20 (17–23)	7 (5–8)	17 (14–21)	51 (38–63)	
70–79	738 (30%)	19 (16–22)	6 (4–7)	16 (12–20)	45 (33–56)	
≥ 80	396 (16%)	17 (14–20)	5 (3–6)	15 (12–18)	39 (29–50)	
		**< 0.001**	**< 0.001**	**< 0.001**	**< 0.001**	*p*‐value
Education						
Below high school	249 (10%)	16 (14–19)	5 (3–6)	14 (11–17)	26 (20–34)	
High school	345 (14%)	18 (15–21)	6 (4–7)	14 (11–18)	37 (29–46)	
Above high school	1896 (76%)	20 (17–23)	7 (5–8)	17 (14–21)	51 (41–62)	
		**< 0.001**	**< 0.001**	**< 0.001**	**< 0.001**	*p*‐value
PIR	2.24 (1.24, 4.28)					
Alcohol						
Nondrinker	767 (31%)	19 (16–22)	6 (5–8)	16 (12–19)	44 (32–56)	
1–5 drinks/month	1205 (49%)	19 (16–22)	6 (5–8)	16 (13–20)	47 (34–59)	
5–10 drinks/month	113 (4.6%)	20 (16–23)	6 (5–8)	18 (15–22)	52 (41–63)	
10+ drinks/month	392 (16%)	20 (17–23)	6 (5–8)	18 (15–22)	52 (41–64)	
		0.491	0.658	**< 0.001**	**< 0.001**	*p*‐value
Smoking						
Nonsmoker	1233 (49%)	20 (16–23)	6 (5–8)	16 (13–20)	49 (35–61)	
Former smoker	917 (37%)	19 (16–22)	6 (5–8)	17 (13–20)	47 (36–59)	
Current smoker	342 (14%)	19 (16–22)	6 (5–8)	16 (13–20)	43 (32–54)	
		**0.048**	0.151	0.085	**< 0.001**	*p*‐value
SBP (mmHg)	131 (119, 143)					
DBP (mmHg)	69 (61, 77)					
Depression						
Yes	216 (8.8%)	19 (15–21)	6 (4–8)	15 (11–19)	39 (28–49)	
No	2250 (91%)	20 (16–22)	6 (5–8)	17 (13–20)	48 (36–60)	
		**0.004**	0.323	**< 0.001**	**< 0.001**	*p*‐value
Stroke						
Yes	161 (6.5%)	18 (15–21)	6 (4–7)	15 (11–18)	38 (27–50)	
No	2326 (94%)	20 (16–22)	6 (5–8)	16 (13–20)	48 (36–60)	
		**< 0.001**	**< 0.001**	**< 0.001**	**< 0.001**	*p*‐value
Diabetes						
Yes	736 (30%)	19 (15–22)	6 (4–7)	15 (12–19)	42 (30–53)	
No	1756 (70%)	20 (16–23)	6 (5–8)	17 (13–21)	49 (37–61)	
		**< 0.001**	**< 0.001**	**< 0.001**	**< 0.001**	*p*‐value
CVD						
Yes	438 (18%)	18 (15–21)	6 (4–7)	16 (12–19)	41 (31–53)	
No	2053 (82%)	20 (16–23)	6 (5–8)	17 (13–20)	48 (36–60)	
		**< 0.001**	**< 0.001**	**< 0.001**	**< 0.001**	*p*‐value
Waist circumference (cm)	101.20 (92.60, 110.60)					
MEDI	3.5 (3.0, 4.5)					
BMI	28.1 (24.8, 32.3)					
BMI group						
Underweight	36 (1.4%)	16 (12–23)	5 (4–7)	14 (12–19)	38 (29–56)	
Normal weight	625 (25%)	20 (16–22)	6 (5–8)	16 (13–20)	47 (35–59)	
Overweight	866 (35%)	19 (16–22)	6 (4–8)	16 (13–20)	47 (35–60)	
Obesity	965 (39%)	20 (17–22)	6 (5–8)	16 (13–20)	47 (35–59)	
		**0.041**	**0.003**	0.142	0.151	*p*‐value

*Note:* Continuous variables are presented as medians with interquartile ranges (IQRs), and categorical variables are expressed as frequencies and percentages [*n* (%)]. Differences between groups were assessed using the Mann–Whitney U test for comparisons between two groups and the Kruskal–Wallis H test for comparisons involving three or more groups. The bold values indicate statistical significance (*p* < 0.05).

Abbreviations: AFT, animal fluency test; BMI, body mass index; CVD, cardiovascular disease; DBP, diastolic blood pressure; DRT, delayed recall test; DSST, Digit Symbol Substitution test; IRT, immediate recall test; MEDI, Mediterranean diet index; PIR, poverty–income ratio; SBP, systolic blood pressure.

### 3.2. Associations of MEDI and BMI Categories With Cognitive Function

Across all three survey‐weighted linear regression models, higher MEDI scores were associated with higher global cognitive function scores (Table 2). In Model 2, MEDI was associated with higher global cognitive function (*β* = 0.22, 95% CI: 0.04 to 0.41, *p* = 0.023). Compared with normal‐weight participants, underweight participants had lower global cognitive function scores in both the unadjusted model and Model 1 (both *p* < 0.05). Participants with obesity had lower global cognitive function scores in Model 1 (*β* = −0.43, 95% CI, −0.83 to −0.03; *p* = 0.035). Additionally, central obesity defined by waist circumference was not significantly associated with global cognitive function in the sensitivity analysis (Supporting Table S1). In the domain‐specific analyses, higher MEDI scores were associated with higher AFT (*β* = 0.43, 95% CI, 0.10 to 0.76, *p* = 0.016) and DSST scores (*β* = 1.30, 95% CI, 0.42 to 2.20, *p* = 0.007), whereas the estimates for IRT and DRT were not statistically significant in Model 2 (Supporting Tables S2–S5). Participants who were underweight had lower IRT (*β* = −2.40, 95% CI, −4.70 to −0.09, *p* = 0.043) and DRT scores (*β* = −1.20, 95% CI, −2.30 to −0.15, *p* = 0.029) than normal‐weight participants. Participants who were overweight had lower AFT scores (*β* = −1.10, 95% CI, −2.10 to −0.07, *p* = 0.038).

**TABLE 2:** Associations of Mediterranean diet index scores and BMI category with global cognitive function: a weighted multivariable linear regression analysis.

	Unadjusted model		Model 1		Model 2	
	*β* (95% CI)	*p* value	*β* (95% CI)	*p* value	*β* (95% CI)	*p* value
MEDI	0.43 (0.26, 0.59)	**< 0.001**	0.34 (0.16, 0.53)	**< 0.001**	0.22 (0.04, 0.41)	**0.023**
BMI category						
Normal weight	—	—	—	—	—	—
Underweight	−2.2 (−3.9, −0.45)	**0.016**	−1.8 (−3.40, −0.30)	**0.021**	−1.5 (−3.1, 0.11)	0.065
Overweight	−0.37 (−0.97, 0.23)	0.2	−0.36 (−0.83, 0.10)	0.12	−0.36 (−0.82, 0.10)	0.11
Obesity	−0.07 (−0.60, 0.47)	0.8	−0.43 (−0.83, −0.03)	**0.035**	−0.11 (−0.56, 0.35)	0.6

*Note:* Data are presented as *β* (95% confidence interval [CI]). Model 1: adjusted for age, sex, and race. Model 2: adjusted for age, sex, race, education, PIR, smoking, SBP, DBP, CVD, stroke, depression, diabetes, and BMI/MEDI. *β*, regression coefficient. The bold values indicate statistical significance (*p* < 0.05).

Abbreviation: CI, confidence interval.

To further characterize the form of the association between MEDI and global cognitive function, RCS analyses were performed. RCS analysis showed a positive, approximately linear association between the MEDI score and global cognitive function (*p* for overall = 0.016; *p* for nonlinearity = 0.976; Figure 2A). Exploratory piecewise‐linear regression estimated a candidate breakpoint at a MEDI score of 2. However, the two‐segment model did not provide a significantly better fit than the single‐slope model (*p* for likelihood ratio test = 0.175, Table 3).

**FIGURE 2:**
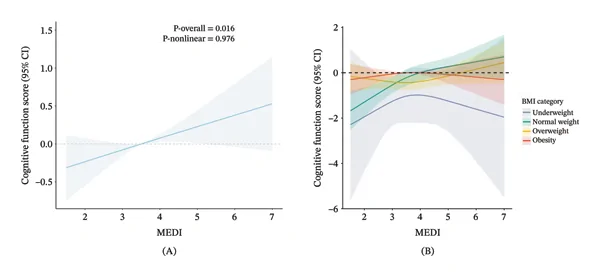
Dose–response relationships between MEDI and global cognitive function. (A) The overall association in the total population is shown, with the solid blue line representing the global cognitive function score and the shaded area indicating the 95% CI. (B) RCS curves stratified by BMI category (underweight, normal weight, overweight, and obesity). The dashed horizontal line at zero represents the reference value. *p* values for the overall association and for nonlinearity were obtained from analysis of variance (ANOVA).

**TABLE 3:** Threshold effect analysis of MEDI on global cognitive function.

	*β* (95% CI)	*p* value
Fitting by standard linear model	0.154 (0.049, 0.258)	**0.004**
Fitting by two‐piecewise linear model		
Inflection point	2	
MEDI < 2	0.912 (−0.193, 2.017)	0.106
MEDI > 2	0.132 (0.023, 0.241)	**0.017**
Likelihood ratio test		0.175

*Note:* The model was adjusted for age, sex, race, education, PIR, smoking, SBP, DBP, BMI, CVD, stroke, depression, and diabetes. The bold values indicate statistical significance (*p* < 0.05).

BMI‐stratified RCS analyses showed that the association between MEDI score and global cognitive function varied across BMI categories (Figure 2B). Among normal‐weight participants, predicted global cognitive function scores increased with increasing MEDI scores, whereas no clear association was apparent in the underweight, overweight, or obesity groups. The overall association was significant only in the normal weight group (*p* for overall < 0.001, Supporting Table S6). Nevertheless, there was no evidence of nonlinearity in any BMI category (*p* for nonlinearity > 0.05). Consistent with the RCS findings, the MEDI × BMI interaction was significant in the linear analysis (*p* for interaction = 0.013, Table 4). Among normal‐weight participants, the MEDI score was associated with a higher global cognitive function score (*β* = 0.475, 95% CI, 0.262 to 0.688; *p* < 0.001). In the sex‐stratified RCS analyses, the curve suggested a positive association between the MEDI score and global cognitive function among men (*p* for overall = 0.020, Supporting Table S7, Supporting Figure S1), whereas no clear trend was apparent among women (*p* for overall = 0.410).

**TABLE 4:** Association between MEDI score and global cognitive function stratified by BMI category.

	*β* (95% CI)	*p* value	*p* for interaction
BMI category			**0.013**
Underweight	0.383 (−1.092, 1.857)	0.582	
Normal weight	0.475 (0.262, 0.688)	**< 0.001**	
Overweight	0.054 (−0.121, 0.229)	0.545	
Obesity	0.024 (−0.147, 0.195)	0.782	

*Note:* Models were adjusted for age, sex, race, education, PIR, smoking, SBP, DBP, CVD, stroke, depression, and diabetes. *p* for interaction refers to the interaction between the MEDI score and BMI category. The bold values indicate statistical significance (*p* < 0.05).

Finally, subgroup analyses further indicated that the association between MEDI and cognitive function was largely consistent across examined demographic and clinical strata. Although the magnitude of the association varied slightly across subgroups, no significant effect modification was detected (*p* for interaction > 0.05), indicating that the association did not differ materially across examined subgroups (Figure 3).

**FIGURE 3:**
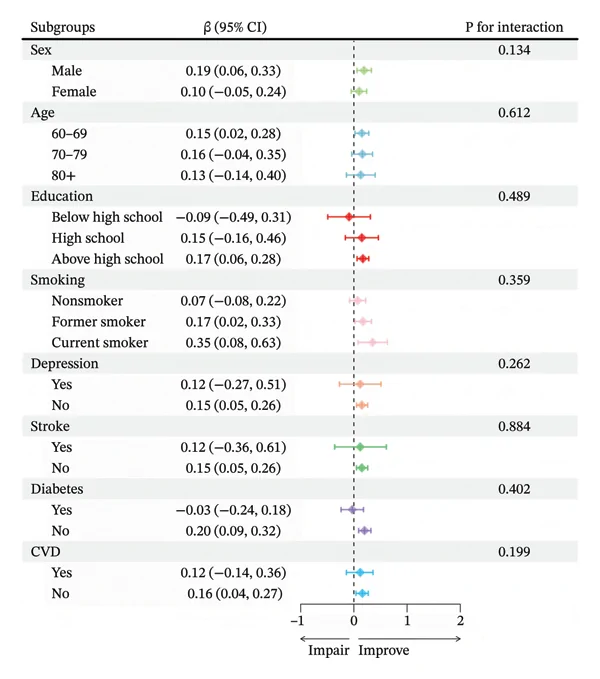
Subgroup analysis of the association between MEDI and global cognitive function. *β* coefficients and 95% CIs were calculated using weighted multivariable linear regression models for each subgroup. Models were adjusted for the same covariates as Model 2, excluding the stratification variable. *p* for interaction was derived from multiplicative interaction terms to assess effect modification across subgroups.

## 4. Discussion

In this nationally representative cross‐sectional study of U.S. adults aged 60 years or older, greater adherence to a Mediterranean dietary pattern was associated with better global cognitive performance after multivariable adjustment. The associations were particularly evident for verbal fluency and processing speed and followed an approximately linear dose–response pattern. However, the association differed across BMI categories, with a positive association observed among normal‐weight participants but no statistically significant association in the other BMI groups. These findings suggest that adiposity may modify the relationship between Mediterranean diet adherence and cognitive function in older adults, potentially reflecting differences in metabolic health.

The current finding that higher MEDI scores were associated with better global cognition is consistent with prospective studies linking Mediterranean‐style diets to lower dementia risk and more favorable cognitive trajectories. Large prospective cohorts, including the U.K. Biobank, the Nurses’ Health Study, and the Health Professionals Follow‐up Study, have linked greater Mediterranean diet adherence to lower dementia risk [7, 8]. Higher adherence has also been associated with better global cognition in the Nurses’ Health Study and slower decline in learning and memory in the Hispanic Community Health Study/Study of Latinos [8, 9]. Observational studies of the MIND diet have reported similar associations [29]. Evidence from randomized trials, however, has been less consistent. PREDIMED cognitive substudies reported benefits of Mediterranean diets supplemented with extra‐virgin olive oil or nuts for global cognition and selected memory or frontal‐executive outcomes, whereas a more recent MIND diet trial showed improvement in both groups but no between‐group difference over 3 years [10, 11, 30]. Accordingly, our cross‐sectional results add to the observational evidence but do not establish that greater Mediterranean diet adherence prevents cognitive decline. Differences in dietary assessment, intervention intensity, baseline nutritional status, comparator diets, cognitive outcomes, and follow‐up duration may explain some of the variation across studies.

Beyond this overall association, domain‐specific analyses showed that higher MEDI scores were associated with better AFT and DSST performance, measures that capture aspects of semantic fluency, executive function, attention, and processing speed. However, the cognitive domains associated with Mediterranean dietary patterns have varied across studies. By comparison, the Hispanic Community Health Study/Study of Latinos reported stronger longitudinal associations with learning and memory than with word fluency or DSST performance [9]. Further biological support comes from neuroimaging and biomarker studies, in which healthier dietary patterns, including Mediterranean‐style diets, have been associated with larger total or regional brain volumes, less atrophy, greater preservation of medial temporal structures, and lower Alzheimer’s disease biomarker burden [31–34]. These observations support biological plausibility but do not identify the mechanisms underlying the AFT and DSST associations in our study.

BMI was associated with selected cognitive outcomes, but the pattern differed across weight categories and cognitive domains. Underweight was associated with poorer immediate and delayed recall, whereas its association with global cognition was attenuated and no longer statistically significant after full adjustment. Large cohort studies have likewise reported a higher risk of dementia among people who are underweight [15, 35]. Pooled longitudinal data indicate, however, that BMI often declines during the preclinical phase of dementia, which may obscure associations between higher midlife BMI and later dementia [12]. Low BMI in older adults may also reflect frailty, loss of lean mass, inadequate energy or protein intake, chronic illness, or weight loss accompanying incipient neurodegeneration. In Model 1, which adjusted for age, sex, and race/ethnicity, obesity was associated with lower global cognitive function; however, the estimate was attenuated after further adjustment. This attenuation may reflect confounding, although adjustment for vascular and metabolic conditions that may lie partly on the pathway linking adiposity to cognitive impairment could also have reduced the estimate. Midlife visceral adiposity has been associated with lower brain volume [36]. In addition, obesity‐related metabolic dysfunction has been linked to greater cerebrovascular burden, structural brain abnormalities, and poorer cognitive performance [37, 38]. Complementing these findings, a 38‐year follow‐up of the Framingham Offspring Study associated obesity at ages 40–49 years with a higher risk of subsequent dementia [39]. Midlife obesity therefore remains a modifiable risk factor in current dementia‐prevention frameworks [3].

RCS analysis further supported a positive, approximately linear association between MEDI score and global cognition. Analyses stratified by BMI showed that the association between MEDI and global cognition varied across BMI categories. In both the linear and spline models, higher MEDI scores were associated with better global cognition among participants with normal weight, whereas no corresponding association was observed in the underweight, overweight, or obesity groups. Among participants with overweight or obesity and metabolic syndrome in PREDIMED‐Plus, greater adherence to an energy‐restricted Mediterranean diet was associated with improvements in memory over 3 years [17]. A subsequent analysis linked greater Mediterranean diet adherence to slower global cognitive decline over 6 years [18]. Differences in study populations, dietary assessment, and follow‐up duration may help account for the variation across studies.

Excess adiposity is often accompanied by insulin resistance, systemic inflammation, and vascular dysfunction, all of which may be relevant to cognitive health [14, 37, 40, 41]. Higher peripheral insulin resistance has been associated with lower regional cerebral glucose metabolism in cognitively normal adults at increased risk for Alzheimer’s disease [42]. In a randomized trial of adults with metabolic syndrome, a Mediterranean‐style dietary intervention reduced insulin resistance and selected inflammatory markers [43]. An isocaloric Mediterranean diet intervention also lowered plasma cholesterol and altered the gut microbiome and systemic metabolome [44]. Gut microbial composition may influence the cardiometabolic associations of Mediterranean diet adherence [45], while signaling along the gut–brain axis provides a potential link between these metabolic responses and cognition [46]. Metabolic health also varies within BMI categories [38], so differences in insulin resistance, fat distribution, and cumulative vascular burden may contribute to the heterogeneity observed here.

These considerations support an integrated approach to cognitive health that combines dietary quality with attention to metabolic and vascular risk. The 2024 Lancet Commission advocates a life‐course approach to modifiable risk factors for dementia [3]. Consistent with this approach, the FINGER and U.S. POINTER trials reported cognitive benefits from multidomain programs incorporating dietary guidance, physical activity, cognitive training, and vascular risk management [4, 47]. SPRINT–MIND also found that intensive blood pressure control reduced the incidence of mild cognitive impairment, although its effect on probable dementia was inconclusive [48]. If replicated, the MEDI × BMI interaction could inform the design of such interventions; it does not justify recommending a Mediterranean dietary pattern only to normal‐weight older adults. Interventions for people with overweight or obesity may need to address dietary quality alongside energy balance, physical activity, and metabolic risk, whereas care for older adults who are underweight should include assessment of dietary adequacy, frailty, sarcopenia, and unintentional weight loss. Future trials could examine whether baseline metabolic health, including insulin resistance, modifies cognitive responses to dietary interventions and whether changes in metabolic health are associated with changes in cognition over time.

This study boasts several strengths, including the use of a well‐characterized, nationally representative sample with a relatively large number of participants. The assessment of multiple cognitive domains allowed the association between MEDI and cognition to be evaluated comprehensively, while the examination of dose–response patterns and potential effect modification provided a more nuanced characterization of this association. Moreover, the concordant results for the interaction between MEDI and BMI in the linear and spline models provided an internal check on the robustness of this finding to model specification. Nevertheless, several limitations of the present study should be noted. First, the cross‐sectional design precludes establishing temporality or causality. Reverse causation cannot be excluded, as poorer cognitive function may influence food choices, appetite, dietary recall, and body weight. Second, dietary intake was assessed using two 24‐h recalls, which may not adequately reflect habitual diet and are susceptible to recall error. The extent of dietary misreporting may also differ according to BMI or cognitive status. Third, BMI and waist circumference cannot distinguish fat mass from lean mass, directly quantify visceral adiposity, or capture sarcopenic obesity, weight history, and unintentional weight loss. Fourth, the small underweight subgroup limited the precision of the estimates in this group, while the multiple domain‐specific and subgroup analyses increased the possibility of chance findings. Finally, the candidate breakpoint and stratified spline findings were exploratory and should be confirmed in longitudinal studies with repeated assessments of diet, adiposity, and cognition.

## 5. Conclusions

In the present study, adherence to a Mediterranean dietary pattern was positively associated with global cognition, including verbal fluency and processing speed. The association differed across BMI categories, with a positive association observed among normal‐weight participants. This BMI‐related heterogeneity highlights the importance of considering adiposity alongside dietary quality in studies of cognitive aging. These findings support longitudinal and interventional studies incorporating repeated assessments of diet, body composition, metabolic health, and cognition to determine whether strategies for preserving cognitive health could be tailored more effectively to older adults’ nutritional and metabolic profiles.

**Nomenclature**
AFT: Animal fluency test
ANOVA: Analysis of variance
BMI: Body mass index
BRIDGE: Building Research in Diet and Cognition
CERAD‐WL: Consortium to Establish a Registry for Alzheimer’s Disease Word Learning
CI: Confidence interval
CVD: Cardiovascular disease
DBP: Diastolic blood pressure
DRT: Delayed recall test
DSST: Digit Symbol Substitution test
FINGER: Finnish Geriatric Intervention Study to Prevent Cognitive Impairment and Disability
FPED: Food Patterns Equivalents Database
IQR: Interquartile range
IRT: Immediate recall test
MEC: Mobile examination center
MEDAS: Mediterranean Diet Adherence Screener
MEDI: Mediterranean diet index
MIND: Mediterranean–Dietary Approaches to Stop Hypertension Intervention for Neurodegenerative Delay
NHANES: National Health and Nutrition Examination Survey
OLS: Ordinary least squares
PIR: Poverty–income ratio
PREDIMED: Prevención con Dieta Mediterránea
RCS: Restricted cubic spline
SBP: Systolic blood pressure
SPRINT–MIND: Systolic Blood Pressure Intervention Trial Memory and Cognition in Decreased Hypertension
USDA: United States Department of Agriculture
U.S. POINTER: U.S. Study to Protect Brain Health Through Lifestyle Intervention to Reduce Risk
VIF: Variance inflation factor
WTDR2D: Dietary 2‐day sample weight

## Author Contributions

Zhaoxing Liu: conceptualization, methodology, formal analysis, investigation, writing–original draft, and writing–review and editing. Mengchen Qin: data curation, data cleaning, data management, and writing–original draft. Chongye Zhao: software, formal analysis, methodology, and writing–review and editing. Ji Ding: data curation, validation, formal analysis, and writing–original draft. Yongkang Li: data curation, validation, formal analysis, and writing–original draft. Weidong Cheng: supervision, funding acquisition, project administration, and writing–review and editing.

## Funding

This study was supported by the National Key Research and Development Program of China (grant no. 2022ZD0211602).

## Disclosure

All authors read and approved the final manuscript.

## Ethics Statement

The National Health and Nutrition Examination Survey (NHANES) 2011–2012 and 2013–2014 protocol was reviewed and approved by the National Center for Health Statistics Research Ethics Review Board (Protocol #2011–17). Written informed consent was obtained from all participants. The present study is a secondary analysis of publicly available, de‐identified NHANES data and therefore did not require additional ethics approval.

## Consent

Please see the Ethics Statement.

## Conflicts of Interest

The authors declare no conflicts of interest.

## Supporting Information

Additional supporting information can be found online in the Supporting Information section.

## Supporting information


**Supporting Information** Supporting Table S1 presents the association between central obesity defined by waist circumference and global cognitive function. Supporting Tables S2–S5 present the associations of MEDI scores and BMI categories with domain‐specific cognitive outcomes. Supporting Tables S6 and S7 present the results of restricted cubic spline analyses stratified by BMI category and sex, respectively. Supporting Figure S1 shows the dose–response relationships between MEDI score and global cognitive function stratified by sex.

## Data Availability

The data that support the findings of this study are available in the National Health and Nutrition Examination Survey (NHANES) website: https://wwwn.cdc.gov/nchs/nhanes/Default.aspx. These data were derived from the following resources available in the public domain: NHANES 2011–2012: Questionnaires, Datasets, and Related Documents, https://wwwn.cdc.gov/Nchs/Nhanes/ContinuousNhanes/Default.aspx?BeginYear=2011. NHANES 2013–2014: Questionnaires, Datasets, and Related Documents, https://wwwn.cdc.gov/nchs/nhanes/ContinuousNhanes/Default.aspx?BeginYear=2013.

## References

[1] GBD 2019 Dementia Forecasting Collaborators , Estimation of the Global Prevalence of Dementia in 2019 and Forecasted Prevalence in 2050: An Analysis for the Global Burden of Disease Study 2019, The Lancet Public Health. (2022) 7, e105–e125. 10.1016/S2468-2667(21)00249-8 PMC8810394 34998485

[2] Aisen P. S. , Cummings J. , Jack C. R. et al., On the Path to 2025: Understanding the Alzheimer’s Disease Continuum, Alzheimer’s Research & Therapy. (2017) 9, no. 1, 10.1186/s13195-017-0283-5. PMC5549378 28793924

[3] Livingston G. , Huntley J. , Liu K. Y. et al., Dementia Prevention, Intervention, and Care: 2024 Report of the Lancet Standing Commission, Lancet. (2024) 404, no. 10452, 572–628, 10.1016/s0140-6736(24)01296-0. 39096926

[4] Baker L. D. , Espeland M. A. , Whitmer R. A. et al., Structured vs Self-Guided Multidomain Lifestyle Interventions for Global Cognitive Function: The US POINTER Randomized Clinical Trial, JAMA. (2025) 334, no. 8, 681–691, 10.1001/jama.2025.12923. PMC12305445 40720610

[5] Guasch-Ferré M. and Willett W. C. , The Mediterranean Diet and Health: A Comprehensive Overview, Journal of Internal Medicine. (2021) 290, no. 3, 549–566, 10.1111/joim.13333. 34423871

[6] Estruch R. , Ros E. , Salas-Salvadó J. et al., Primary Prevention of Cardiovascular Disease With a Mediterranean Diet Supplemented With Extra-Virgin Olive Oil or Nuts, New England Journal of Medicine. (2018) 378, no. 25, 10.1056/nejmoa1800389. 29897866

[7] Shannon O. M. , Ranson J. M. , Gregory S. et al., Mediterranean Diet Adherence is Associated With Lower Dementia Risk, Independent of Genetic Predisposition: Findings From the UK Biobank Prospective Cohort Study, BMC Medicine. (2023) 21, no. 1, 10.1186/s12916-023-02772-3. PMC10012551 36915130

[8] Liu Y. , Gu X. , Li Y. et al., Interplay of Genetic Predisposition, Plasma Metabolome and Mediterranean Diet in Dementia Risk and Cognitive Function, Nature Medicine. (2025) 31, no. 11, 3791–3800, 10.1038/s41591-025-03891-5. PMC12618253 40855194

[9] Moustafa B. , Trifan G. , Isasi C. R. et al., Association of Mediterranean Diet With Cognitive Decline Among Diverse Hispanic or Latino Adults From the Hispanic Community Health Study/Study of Latinos, JAMA Network Open. (2022) 5, no. 7, 10.1001/jamanetworkopen.2022.21982. PMC9284337 35834250

[10] Valls-Pedret C. , Sala-Vila A. , Serra-Mir M. et al., Mediterranean Diet and Age-Related Cognitive Decline: A Randomized Clinical Trial, JAMA Internal Medicine. (2015) 175, no. 7, 1094–1103, 10.1001/jamainternmed.2015.1668. 25961184

[11] Barnes L. L. , Dhana K. , Liu X. et al., Trial of the Mind Diet for Prevention of Cognitive Decline in Older Persons, New England Journal of Medicine. (2023) 389, no. 7, 602–611, 10.1056/nejmoa2302368. PMC10513737 37466280

[12] Kivimäki M. , Luukkonen R. , Batty G. D. et al., Body Mass Index and Risk of Dementia: Analysis of Individual-Level Data From 1.3 Million Individuals, Alzheimer’s & Dementia: Journal of Alzheimer’s Association. (2018) 14, no. 5, 601–609, 10.1016/j.jalz.2017.09.016. PMC5948099 29169013

[13] Whitmer R. A. , Gustafson D. R. , Barrett-Connor E. , Haan M. N. , Gunderson E. P. , and Yaffe K. , Central Obesity and Increased Risk of Dementia More Than Three Decades Later, Neurology. (2008) 71, no. 14, 1057–1064, 10.1212/01.wnl.0000306313.89165.ef. 18367704

[14] Qureshi D. , Topiwala A. , Al A. S. U. , Allen N. E. , Kuźma E. , and Littlejohns T. J. , Association of Metabolic Syndrome With Neuroimaging and Cognitive Outcomes in the UK Biobank, Diabetes Care. (2024) 47, no. 8, 1415–1423, 10.2337/dc24-0537. PMC11272984 38894691

[15] Qizilbash N. , Gregson J. , Johnson M. E. et al., BMI and Risk of Dementia in Two Million People Over Two Decades: A Retrospective Cohort Study, Lancet Diabetes and Endocrinology. (2015) 3, no. 6, 431–436, 10.1016/s2213-8587(15)00033-9. 25866264

[16] Nishi S. K. , Babio N. , Gómez-Martínez C. et al., Mediterranean, DASH, and MIND Dietary Patterns and Cognitive Function: The 2-Year Longitudinal Changes in an Older Spanish Cohort, Frontiers in Aging Neuroscience. (2021) 13, 10.3389/fnagi.2021.782067. PMC8710807 34966270

[17] Soldevila-Domenech N. , Forcano L. , Vintró-Alcaraz C. et al., Interplay Between Cognition and Weight Reduction in Individuals Following a Mediterranean Diet: Three-Year Follow-Up of the Predimed-Plus Trial, Clinical Nutrition. (2021) 40, no. 9, 5221–5237, 10.1016/j.clnu.2021.07.020. 34474192

[18] Ni J. , Hernández-Cacho A. , Nishi S. K. et al., Mediterranean Diet, Gut Microbiota, and Cognitive Decline in Older Adults With Obesity/Overweight and Metabolic Syndrome: A Prospective Cohort Study, BMC Medicine. (2025) 23, no. 1, 10.1186/s12916-025-04488-y. PMC12667113 41327300

[19] McLeod A. , Lamar M. , Schiffer L. et al., Long-Term Outcomes of a Mediterranean Diet With or Without Calorie Restriction on Cognition and Cardiometabolic Health: The Building Research in Diet and Cognition Trial, Preventive Medicine Reports. (2025) 55, 10.1016/j.pmedr.2025.103099. PMC12207229 40589552

[20] Johnson C. L. , Dohrmann S. M. , Burt V. L. et al., National Health and Nutrition Examination Survey: Sample Design, 2011–2014, Vital Health Statistics. (2014) 2, 1–33. 25569458

[21] Liu S. and Chen X. , MedDiet Adherence Score for the Association Between Inflammatory Markers and Cognitive Performance in the Elderly: A Study of the NHANES 2011–2014, BMC Geriatrics. (2022) 22, no. 1, 10.1186/s12877-022-03140-1. PMC9215079 35729501

[22] Booranasuksakul U. , Macdonald I. A. , Stephan B. C. M. , and Siervo M. , Body Composition, Sarcopenic Obesity, and Cognitive Function in Older Adults: Findings From the National Health and Nutrition Examination Survey (NHANES) 1999–2002 and 2011–2014, Journal of the American Nutrition Association. (2024) 43, no. 6, 539–552, 10.1080/27697061.2024.2333310. 38564377

[23] Moshfegh A. J. , Rhodes D. G. , Baer D. J. et al., The US Department of Agriculture Automated Multiple-Pass Method Reduces Bias in the Collection of Energy Intakes, American Journal of Clinical Nutrition. (2008) 88, no. 2, 324–332, 10.1093/ajcn/88.2.324. 18689367

[24] Ahluwalia N. , Dwyer J. , Terry A. , Moshfegh A. , and Johnson C. , Update on NHANES Dietary Data: Focus on Collection, Release, Analytical Considerations, and Uses to Inform Public Policy, Advances in Nutrition. (2016) 7, no. 1, 121–134, 10.3945/an.115.009258. PMC4717880 26773020

[25] Zhan J. J. , Hodge R. A. , Dunlop A. L. et al., Dietaryindex: A User-Friendly and Versatile R Package for Standardizing Dietary Pattern Analysis in Epidemiological and Clinical Studies, American Journal of Clinical Nutrition. (2024) 120, no. 5, 1165–1174, 10.1016/j.ajcnut.2024.08.021. PMC11600030 39182618

[26] Schröder H. , Fitó M. , Estruch R. et al., A Short Screener is Valid for Assessing Mediterranean Diet Adherence Among Older Spanish Men and Women, Journal of Nutrition. (2011) 141, no. 6, 1141–1145, 10.3945/jn.110.135566. 21508208

[27] Herbert B. M. , Johnson A. E. , Paasche-Orlow M. K. , Brooks M. M. , and Magnani J. W. , Disparities in Reporting a History of Cardiovascular Disease Among Adults With Limited English Proficiency and Angina, JAMA Network Open. (2021) 4, no. 12, 10.1001/jamanetworkopen.2021.38780. PMC8672228 34905003

[28] Chen T.-C. , Parker J. D. , Clark J. et al., National Health and Nutrition Examination Survey: Estimation Procedures, 2011-2014, Vital Health Statistics. (2018) 2, 1–26. 29775431

[29] Morris M. C. , Tangney C. C. , Wang Y. et al., Mind Diet Slows Cognitive Decline WITH Aging, Alzheimer’s & Dementia: Journal of Alzheimer’s Association. (2015) 11, no. 9, 1015–1022, 10.1016/j.jalz.2015.04.011. PMC4581900 26086182

[30] Martínez-Lapiscina E. H. , Clavero P. , Toledo E. et al., Mediterranean Diet Improves Cognition: The Predimed-Navarra Randomised Trial, Journal of Neurology Neurosurgery and Psychiatry. (2013) 84, no. 12, 1318–1325, 10.1136/jnnp-2012-304792. 23670794

[31] Gu Y. , Brickman A. M. , Stern Y. et al., Mediterranean Diet and Brain Structure in a Multiethnic Elderly Cohort, Neurology. (2015) 85, no. 20, 1744–1751, 10.1212/wnl.0000000000002121. PMC4653103 26491085

[32] Luciano M. , Corley J. , Cox S. R. et al., Mediterranean-Type Diet and Brain Structural Change From 73 to 76 Years in a Scottish Cohort, Neurology. (2017) 88, no. 5, 449–455, 10.1212/wnl.0000000000003559. PMC5278943 28053008

[33] Croll P. H. , Voortman T. , Ikram M. A. et al., Better Diet Quality Relates to Larger Brain Tissue Volumes: The Rotterdam Study, Neurology. (2018) 90, no. 24, e2166–e2173, 10.1212/wnl.0000000000005691. 29769374

[34] Ballarini T. , Melo van Lent D. , Brunner J. et al., Mediterranean Diet, Alzheimer Disease Biomarkers and Brain Atrophy in Old Age, Neurology. (2021) 96, no. 24, e2921–e2932, 10.1212/wnl.0000000000012067. PMC8253566 33952652

[35] Lee C. M. , Woodward M. , Batty G. D. et al., Association of Anthropometry and Weight Change With Risk of Dementia and Its Major Subtypes: A Meta-Analysis Consisting 2.8 Million Adults WITH 57 294 Cases of Dementia, Obesity Reviews: International Journal of Obesity. (2020) 21, no. 4, 10.1111/obr.12989. PMC7079047 31898862

[36] Debette S. , Beiser A. , Hoffmann U. et al., Visceral Fat is Associated with Lower Brain Volume in Healthy middle-aged Adults, Annals of Neurology. (2010) 68, no. 2, 136–144, 10.1002/ana.22062. PMC2933649 20695006

[37] Morys F. , Dadar M. , and Dagher A. , Association Between Midlife Obesity and its Metabolic Consequences, Cerebrovascular Disease, and Cognitive Decline, Journal of Clinical Endocrinology and Metabolism. (2021) 106, no. 10, e4261–e4274, 10.1210/clinem/dgab135. PMC8475210 33677592

[38] Angoff R. , Himali J. J. , Maillard P. et al., Relations of Metabolic Health and Obesity to Brain Aging in Young to Middle-Aged Adults, Journal of the American Heart Association. (2022) 11, no. 6, 10.1161/jaha.121.022107. PMC9075324 35229662

[39] Li J. , Joshi P. , Ang T. F. A. et al., Mid-to Late-Life Body Mass Index and Dementia Risk: 38 Years of Follow-Up of the Framingham Study, American Journal of Epidemiology. (2021) 190, no. 12, 2503–2510, 10.1093/aje/kwab096. PMC8796797 33831181

[40] Powell-Wiley T. M. , Poirier P. , Burke L. E. et al., Obesity and Cardiovascular Disease: A Scientific Statement From the American Heart Association, Circulation. (2021) 143, no. 21, e984–e1010, 10.1161/cir.0000000000000973. PMC8493650 33882682

[41] Tai X. Y. , Veldsman M. , Lyall D. M. et al., Cardiometabolic Multimorbidity, Genetic Risk, and Dementia: A Prospective Cohort Study, The Lancet. Healthy Longevity. (2022) 3, no. 6, e428–e436, 10.1016/s2666-7568(22)00117-9. PMC9184258 35711612

[42] Willette A. A. , Bendlin B. B. , Starks E. J. et al., Association of Insulin Resistance With Cerebral Glucose Uptake in Late Middle-Aged Adults at Risk for Alzheimer Disease, JAMA Neurology. (2015) 72, no. 9, 1013–1020, 10.1001/jamaneurol.2015.0613. PMC4570876 26214150

[43] Esposito K. , Marfella R. , Ciotola M. et al., Effect of a Mediterranean-Style Diet on Endothelial Dysfunction and Markers of Vascular Inflammation in the Metabolic Syndrome: A Randomized Trial, JAMA. (2004) 292, 1441–1446. 10.1001/jama.292.12.1440 15383514

[44] Meslier V. , Laiola M. , Roager H. M. et al., Mediterranean Diet Intervention in Overweight and Obese Subjects Lowers Plasma Cholesterol and Causes Changes in the Gut Microbiome and Metabolome Independently of Energy Intake, Gut. (2020) 69, no. 7, 1258–1268, 10.1136/gutjnl-2019-320438. PMC7306983 32075887

[45] Wang D. D. , Nguyen L. H. , Li Y. et al., The Gut Microbiome Modulates the Protective Association Between a Mediterranean Diet and Cardiometabolic Disease Risk, Nature Medicine. (2021) 27, no. 2, 333–343, 10.1038/s41591-020-01223-3. PMC8186452 33574608

[46] Agirman G. , Yu K. B. , and Hsiao E. Y. , Signaling Inflammation Across the gut-brain Axis, Science. (2021) 374, no. 6571, 1087–1092, 10.1126/science.abi6087. 34822299

[47] Ngandu T. , Lehtisalo J. , Solomon A. et al., A 2 Year Multidomain Intervention of Diet, Exercise, Cognitive Training, and Vascular Risk Monitoring Versus Control to Prevent Cognitive Decline in at-risk Elderly People (FINGER): a Randomised Controlled Trial, Lancet. (2015) 385, no. 9984, 2255–2263, 10.1016/s0140-6736(15)60461-5. 25771249

[48] Sprint Mind Investigators For The Sprint Research Group , Williamson J. D. , Pajewski N. M. et al., Effect of Intensive vs Standard Blood Pressure Control on Probable Dementia: A Randomized Clinical Trial, JAMA. (2019) 321, 553–561. 10.1001/jama.2018.21442 PMC6439590 30688979

