# Supplementary material for: Mediterranean Diet Adherence and Cognitive Function in Older Adults: Heterogeneity in the Association Across BMI Categories
Source: Int J Endocrinol. 2026 Oct 28;2026:7060250. doi: 10.1155/ije/7060250 (PMC13715489; doi:10.1155/ije/7060250)
Supplement: Supplementary file 1 [file IJE-2026-7060250-s001.zip › Supplementary Figure S1.pdf]

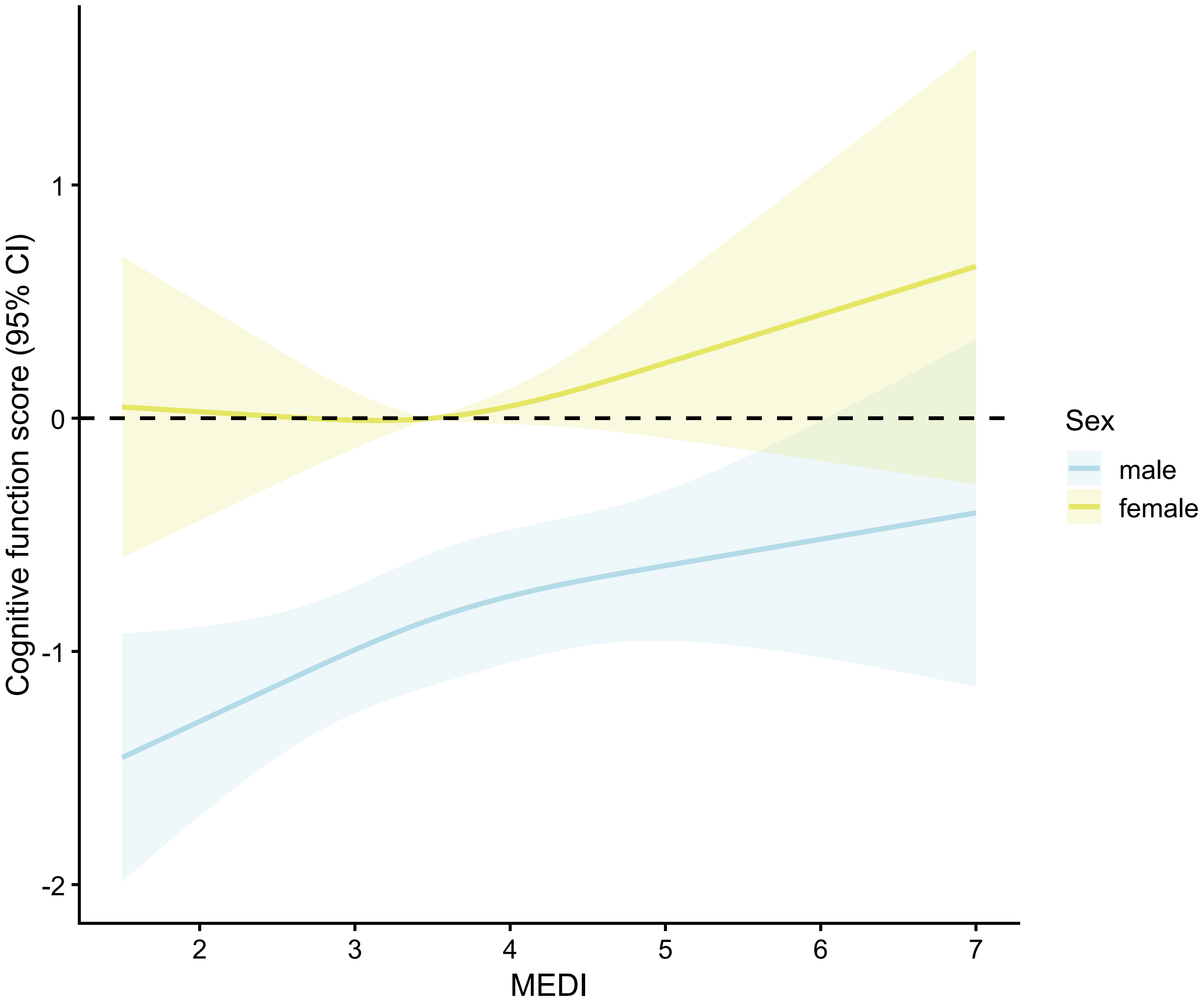

Cognitive function score (95% CI)
1
0
-1
-2
2
3
4
5
6
7
MEDI
Sex
male
female
