# Supplementary material for: Mediterranean Diet Adherence and Cognitive Function in Older Adults: Heterogeneity in the Association Across BMI Categories
Source: Int J Endocrinol. 2026 Oct 28;2026:7060250. doi: 10.1155/ije/7060250 (PMC13715489; doi:10.1155/ije/7060250)
Supplement: Supplementary file 1 [file IJE-2026-7060250-s001.zip › Supplementary Table S1.docx]

**Supplementary Table S1 | Associations of Waist circumference with global cognitive function: a weighted multivariable linear regression analysis.**

|  | Unadjusted Model | | Model 1 | | Model 2 | |
| --- | --- | --- | --- | --- | --- | --- |
|  | β(95%CI) | *P* value | β(95%CI) | *P* value | β(95%CI) | *P* value |
| **Waist circumference** |  |  |  |  |  |  |
| Normal waist circumference | - | - | - | - | - | - |
| Central Obesity | 0.24 (-0.22, 0.70) | 0.3 | -0.15 (-0.52, 0.21) | 0.4 | 0.00 (-0.38, 0.37) | >0.9 |

Data are presented as β (95% confidence interval [CI]).

Model 1: adjusted for age, sex, and race.

Model 2: adjusted for age, sex, race, education, smoking, PIR, SBP, DBP, CVD, stroke, depression, diabetes, and MEDI.

β, regression coefficient; CI, confidence interval.
