# Supplementary material for: Mediterranean Diet Adherence and Cognitive Function in Older Adults: Heterogeneity in the Association Across BMI Categories
Source: Int J Endocrinol. 2026 Oct 28;2026:7060250. doi: 10.1155/ije/7060250 (PMC13715489; doi:10.1155/ije/7060250)
Supplement: Supplementary file 1 [file IJE-2026-7060250-s001.zip › Supplementary Table S2-S5.docx]

**Supplementary Table S2 | Associations of MEDI score and BMI categories with immediate recall test (IRT): a weighted multivariable linear regression analysis**

|  | Unadjusted Model | | Model 1 | | Model 2 | |
| --- | --- | --- | --- | --- | --- | --- |
|  | β(95%CI) | *P* value | β(95%CI) | *P* value | β(95%CI) | *P* value |
| **MEDI** | 0.36 (0.04, 0.68) | **0.027** | 0.26 (-0.08, 0.60) | 0.13 | 0.15 (-0.19, 0.48) | 0.4 |
| **BMI** |  |  |  |  |  |  |
| Normal weight | - | - | - | - | - | - |
| Underweight | -3.2 (-5.8, -0.63) | **0.017** | -2.9 (-5.3, -0.50) | **0.020** | -2.4 (-4.7, -0.09) | **0.043** |
| Overweight | -0.58 (-1.3, 0.15) | 0.11 | -0.45 (-1.1, 0.16) | 0.14 | -0.46 (-1.1, 0.17) | 0.14 |
| Obesity | -0.01 (-0.78, 0.76) | >0.9 | -0.37 (-1.1, 0.32) | 0.3 | -0.10 (-0.83, 0.64) | 0.8 |

Data are presented as β (95% confidence interval [CI]).

Model 1: adjusted for age, sex, and race.

Model 2: adjusted for age, sex, race, education, PIR, smoking, SBP, DBP, CVD, stroke, depression, diabetes, and BMI/MEDI.

β, regression coefficient; CI, confidence interval.

**Supplementary Table S3 | Associations of MEDI score and BMI categories with delayed recall test (DRT): a weighted multivariable linear regression analysis.**

|  | Unadjusted Model | | Model 1 | | Model 2 | |
| --- | --- | --- | --- | --- | --- | --- |
|  | β(95%CI) | *P* value | β(95%CI) | *P* value | β(95%CI) | *P* value |
| **MEDI** | 0.20 (0.06, 0.33) | **0.005** | 0.13 (-0.02, 0.28) | 0.10 | 0.08 (-0.08, 0.25) | 0.3 |
| **BMI** |  |  |  |  |  |  |
| Normal weight | - | - | - | - | - | - |
| Underweight | -1.4 (-2.3, -0.49) | **0.004** | -1.2 (-2.1, -0.34) | **0.008** | -1.2 (-2.3, -0.15) | **0.029** |
| Overweight | -0.26 (-0.69, 0.16) | 0.2 | -0.17 (-0.53, 0.19) | 0.3 | -0.16 (-0.54, 0.23) | 0.4 |
| Obesity | -0.01 (-0.33, 0.31) | >0.9 | -0.12 (-0.42, 0.17) | 0.4 | 0.00 (-0.32, 0.32) | >0.9 |

Data are presented as β (95% confidence interval [CI]).

Model 1: adjusted for age, sex, and race.

Model 2: adjusted for age, sex, race, education, PIR, smoking, SBP, DBP, CVD, stroke, depression, diabetes, and BMI/MEDI.

β, regression coefficient; CI, confidence interval.

**Supplementary Table S4 | Associations of MEDI score and BMI categories with animal fluency test (AFT): a weighted multivariable linear regression analysis.**

|  | Unadjusted Model | | Model 1 | | Model 2 | |
| --- | --- | --- | --- | --- | --- | --- |
|  | β(95%CI) | *P* value | β(95%CI) | *P* value | β(95%CI) | *P* value |
| **MEDI** | 0.63 (0.32, 0.94) | **<0.001** | 0.59 (0.29, 0.90) | **<0.001** | 0.43 (0.10, 0.76) | **0.016** |
| **BMI** |  |  |  |  |  |  |
| Normal weight | - | - | - | - | - | - |
| Underweight | -2.3 (-5.4, 0.82) | 0.14 | -1.6 (-4.0, 0.73) | 0.2 | -0.94 (-3.4, 1.5) | 0.4 |
| Overweight | -0.55 (-1.6, 0.56) | 0.3 | -0.93 (-1.9, 0.03) | 0.057 | -1.1 (-2.1, -0.07) | **0.038** |
| Obesity | -0.25 (-1.3, 0.82) | 0.6 | -0.89 (-1.8, 0.06) | 0.065 | -0.65 (-1.8, 0.51) | 0.2 |

Data are presented as β (95% confidence interval [CI]).

Model 1: adjusted for age, sex, and race.

Model 2: adjusted for age, sex, race, education, PIR, smoking, SBP, DBP, CVD, stroke, depression, diabetes, and BMI/MEDI.

β, regression coefficient; CI, confidence interval.

**Supplementary Table S5 | Associations of MEDI score and BMI categories with digit symbol substitution test (DSST) a weighted multivariable linear regression analysis.**

|  | Unadjusted Model | | Model 1 | | Model 2 | |
| --- | --- | --- | --- | --- | --- | --- |
|  | β(95%CI) | *P* value | β(95%CI) | *P* value | β(95%CI) | *P* value |
| **MEDI** | 2.4 (1.5, 3.4) | **<0.001** | 1.9 (1.0, 2.8) | **<0.001** | 1.3 (0.42, 2.2) | **0.007** |
| **BMI** |  |  |  |  |  |  |
| Normal weight | - | - | - | - | - | - |
| Underweight | -7.7 (-16, 0.85) | 0.076 | -5.2 (-13, 2.2) | 0.2 | -3.8 (-11, 3.8) | 0.3 |
| Overweight | -0.47 (-3.3, 2.4) | 0.7 | -0.02 (-2.0, 2.0) | >0.9 | 0.13 (-1.9, 2.2) | 0.9 |
| Obesity | -0.25 (-3.4, 2.9) | 0.9 | -1.1 (-3.4, 1.1) | 0.3 | 0.57 (-2.1, 3.2) | 0.6 |

Data are presented as β (95% confidence interval [CI]).

Model 1: adjusted for age, sex, and race.

Model 2: adjusted for age, sex, race, education, PIR, smoking, SBP, DBP, CVD, stroke, depression, diabetes, and BMI/MEDI.

β, regression coefficient; CI, confidence interval.
