# Supplementary material for: Mediterranean Diet Adherence and Cognitive Function in Older Adults: Heterogeneity in the Association Across BMI Categories
Source: Int J Endocrinol. 2026 Oct 28;2026:7060250. doi: 10.1155/ije/7060250 (PMC13715489; doi:10.1155/ije/7060250)
Supplement: Supplementary file 1 [file IJE-2026-7060250-s001.zip › Supplementary Table S6.docx]

**Supplementary Table S6 | Restricted cubic spline analysis of the association between MEDI score and global cognitive function by BMI.**

|  | *P*-overall | | *P*-nonlinear | | *P* for interaction |
| --- | --- | --- | --- | --- | --- |
| **BMI** | |  | |  | **0.014** |
| Underweight | | 0.857 | | 0.888 |  |
| Normal weight | | **<0.001** | | 0.135 |  |
| Overweight | | 0.346 | | 0.185 |  |
| Obesity | | 0.725 | | 0.451 |  |

Restricted cubic spline models with three knots were used to evaluate the association between MEDI score and global cognitive function across BMI categories.

Models were adjusted for age, sex, race, education, PIR, smoking, SBP, DBP, CVD, stroke, depression, and diabetes.

P-overall represents the joint test of the linear and nonlinear spline components, whereas P-nonlinear evaluates departure from linearity. P for interaction evaluates whether the MEDI–cognitive function association differs across BMI categories.
